# Second Primary Lung Cancer Among Lung Cancer Survivors Who Never Smoked

**DOI:** 10.1001/jamanetworkopen.2023.43278

**Published:** 2023-11-15

**Authors:** Eunji Choi, Chloe C. Su, Julie T. Wu, Jacqueline V. Aredo, Joel W. Neal, Ann N. Leung, Leah M. Backhus, Natalie S. Lui, Loïc Le Marchand, Daniel O. Stram, Su-Ying Liang, Iona Cheng, Heather A. Wakelee, Summer S. Han

**Affiliations:** 1 Quantitative Sciences Unit, Stanford University School of Medicine, Stanford, California; 2 Department of Epidemiology and Population Health, Stanford University School of Medicine, Stanford, California; 3 Department of Medicine, Stanford University School of Medicine, Stanford, California; 4 Department of Medicine, University of California, San Francisco; 5 Stanford Cancer Institute, Stanford, California; 6 Department of Radiology, Stanford University School of Medicine, Stanford, California; 7 Department of Cardiothoracic Surgery, Stanford University School of Medicine, Stanford, California; 8 Cancer Epidemiology Program, University of Hawaii Cancer Center, Honolulu; 9 Department of Preventive Medicine, Keck School of Medicine, University of Southern California, Los Angeles; 10 Sutter Health, Palo Alto Medical Foundation Research Institute, Palo Alto, California; 11 Department of Epidemiology and Biostatistics, University of California, San Francisco; 12 Department of Neurosurgery, Stanford University School of Medicine, Stanford, California

## Abstract

**Importance:**

Lung cancer among never-smokers accounts for 25% of all lung cancers in the US; recent therapeutic advances have improved survival among patients with initial primary lung cancer (IPLC), who are now at high risk of developing second primary lung cancer (SPLC). As smoking rates continue to decline in the US, it is critical to examine more closely the epidemiology of lung cancer among patients who never smoked, including their risk for SPLC.

**Objective:**

To estimate and compare the cumulative SPLC incidence among lung cancer survivors who have never smoked vs those who have ever smoked.

**Design, Setting, and Participants:**

This population-based prospective cohort study used data from the Multiethnic Cohort Study (MEC), which enrolled participants between April 18, 1993, and December 31, 1996, with follow-up through July 1, 2017. Eligible individuals for this study were aged 45 to 75 years and had complete smoking data at baseline. These participants were followed up for IPLC and further SPLC development through the Surveillance, Epidemiology, and End Results registry. The data were analyzed from July 1, 2022, to January 31, 2023.

**Exposures:**

Never-smoking vs ever-smoking exposure at MEC enrollment.

**Main Outcomes and Measures:**

The study had 2 primary outcomes: (1) 10-year cumulative incidence of IPLC in the entire study cohort and 10-year cumulative incidence of SPLC among patients with IPLC and (2) standardized incidence ratio (SIR) (calculated as the SPLC incidence divided by the IPLC incidence) by smoking history.

**Results:**

Among 211 414 MEC participants, 7161 (3.96%) developed IPLC over 4 038 007 person-years, and 163 (2.28%) developed SPLC over 16 470 person-years. Of the participants with IPLC, the mean (SD) age at cohort enrollment was 63.6 (7.7) years, 4031 (56.3%) were male, and 3131 (43.7%) were female. The 10-year cumulative IPLC incidence was 2.40% (95% CI, 2.31%-2.49%) among ever-smokers, which was 7 times higher than never-smokers (0.34%; 95% CI, 0.30%-0.37%). However, the 10-year cumulative SPLC incidence following IPLC was as high among never-smokers (2.84%; 95% CI, 1.50%-4.18%) as ever-smokers (2.72%; 95% CI, 2.24%-3.20%), which led to a substantially higher SIR for never-smokers (14.50; 95% CI, 8.73-22.65) vs ever-smokers (3.50; 95% CI, 2.95-4.12).

**Conclusions and Relevance:**

The findings indicate that SPLC risk among lung cancer survivors who never smoked is as high as among those with IPLC who ever-smoked, highlighting the need to identify risk factors for SPLC among patients who never smoked and to develop a targeted surveillance strategy.

## Key Points

**Question:**

What is the risk of developing second primary lung cancer (SPLC) among lung cancer survivors who have never smoked?

**Findings:**

In this cohort study of 211 414 participants, the cumulative 10-year incidence of initial primary lung cancer (IPLC) among ever-smokers was 7 times higher than for never-smokers. However, the cumulative SPLC incidence following IPLC was as high among lung cancer survivors who never smoked as those who ever smoked.

**Meaning:**

These findings highlight the need to identify risk factors for SPLC among lung cancer survivors who never smoked and to develop a targeted surveillance strategy.

## Teaser

This cohort study characterizes and quantifies second primary lung cancer incidence and disease burden among lung cancer survivors who never vs ever smoked.

## Introduction

Lung cancer remains the leading cause of cancer-related mortality in the US, causing more deaths than breast, prostate, and colon cancer combined.^1^ While smoking is the predominant risk factor, approximately one-quarter of patients worldwide develop lung cancer without a smoking history.^2,3,4^ Although the risk factors for lung cancer among never-smokers have not been thoroughly examined, prior studies have associated factors such as radon exposure,^5^ secondhand smoke,^6^ and genetic susceptability^7^ with lung cancer in this patient population. Patients who have never smoked make up 10% to 15% of the lung cancer population in the US and Europe and up to 40% in Asia.^8,9,10^ As smoking rates continue to decrease,^11^ it is critical to closely examine the epidemiology of lung cancer among patients without a smoking history.

Concurrently, second primary lung cancer (SPLC) is an increasing public health concern.^12^ The number of lung cancer survivors has been increasing with early detection through screening and therapeutic advances. However, lung cancer survivors have a 4- to 6-times higher risk of developing SPLC compared with the risk of developing initial primary lung cancer (IPLC) in the general population^13^; the cumulative incidence of SPLC has increased continuously without plateau.^12^ Although prior studies have examined SPLC risk factors,^14,15,16^ such as surgical resection for IPLC^15^ and tobacco smoking,^14,16^ and have developed SPLC prediction models,^17,18^ they lack insight into the patterns of SPLC incidence specifically among lung cancer survivors who never smoked. A few single-institution studies for SPLC have included never-smokers, but the numbers of cases were limited,^16,19^ and, to our knowledge, no prior studies have characterized the patterns of SPLC incidence among never-smoking survivors using prospective, population-based cohort data. In this study, we aimed to characterize and quantify SPLC incidence and disease burden among lung cancer survivors who have never vs ever smoked from a large, population-based cohort with long-term prospective follow-up.

## Methods

### Study Population

For this cohort study, data were derived from the Multiethnic Cohort Study (MEC), which includes a large, ethnically diverse cohort of 214 862 healthy adults in California and Hawaii. The MEC was established to examine key factors associated with cancer risk among adults aged 45 to 75 years at cohort enrollment (April 18, 1993, to December 31, 1996) across different racial groups with the following racial and ethnic distribution: African American (16.2%), Japanese American (26.4%), Latino (22.1%), Native Hawaiian (6.7%), White (23.0%), and other (5.7%).^20^ At enrollment, participant characteristics were collected through a baseline self-report questionnaire that measured dietary and nondietary risk factors, including smoking, alcohol consumption, and physical activity. The receipt of a questionnaire was considered as consent to participate in the MEC by the institutional review boards of the University of Hawaii and the University of Southern California; all participating sites received a waiver of consent per institutional review board guidelines due to the absence of processes requiring written consent, the sharing of deidentified data, and the expectation of no more than minimal risk of harm. This study followed the Strengthening the Reporting of Observational Studies in Epidemiology (STROBE) reporting guideline.

The incidence of IPLC and SPLC was identified through linkage to 2 state-level Surveillance, Epidemiology, and End Results registries, the Hawaii Tumor Registry and the California State Cancer Registry, through July 1, 2017. In this study, we excluded participants with missing smoking status (n = 3448 [1.6%]) in order to conduct a complete case analysis.

### SPLC Definition

To accurately ascertain SPLC cases, we applied the most widely used clinical criteria by Martini and Melamed.^21^ Per the Martini and Melamed criteria, new primary lung cancers (ie, SPLC) should meet at least 1 of the following conditions: (1) have different histology from that of the IPLC, (2) have at least 2 years of a disease-free interval from the time of IPLC diagnosis, or (3) arise in distinct lobes or lungs with no evidence of common lymphatics and extrapulmonary metastases at diagnosis.^21^ To reduce the potential misclassification of recurrent or metastatic cases as SPLC in registry data, we used only the first 2 definitions.^17,22^

### Study Outcomes

The study had 2 primary outcomes. The first was the cumulative incidence of IPLC and SPLC. To account for the competing risk of death, we applied the Aalen-Johansen estimator^23,24,25^ to calculate the cumulative incidence of IPLC in the entire cohort and the cumulative incidence of SPLC among patients with IPLC. The Aalen-Johansen estimator is a nonparametric estimator of the cumulative incidence function for a disease of interest using time-to-event data under competing risk.^23,24,25^ The cumulative incidence function is modeled separately for each cause and event (including a competing event) using the overall survival information and the history of events and censoring over time. This method estimates the cumulative incidence of the disease of interest by removing patients who experience a competing event from the risk set, recognizing that the event of interest can no longer happen after the occurrence of a competing event. The cumulative incidence was stratified by smoking history.^26^

The second outcome was the standardized incidence ratio (SIR) by smoking history, calculated as the SPLC incidence (observed SPLC cases over the person-years of the patients with IPLC) divided by the IPLC incidence (observed IPLC cases over the person-years of the entire cohort), which follows the National Cancer Institute’s multiple primary-SIR (MP-SIR) approach.^27^ The MP-SIR describes the ratio of the incidence of new primary cancer in a population with initial primary cancer vs the incidence of initial primary cancer in the general population. We used the MP-SIR to calculate the incidence of new primary lung cancer (ie, SPLC cases) among patients who already had an IPLC diagnosis (numerator) compared with the incidence of new primary lung cancer (ie, IPLC cases) in the general population (denominator). We can then interpret the resulting MP-SIR estimate to be the comparative level of incidence for new primary lung cancers among patients already diagnosed with IPLC vs those without IPLC (ie, the general population). The 95% CI of the SIR was calculated using Byar approximation according to the MP-SIR method.^27^ Given the common and error-prone issue of interpreting SIRs compared with risk ratios (eg, hazard ratio, relative risk ratio),^28^ we included a comparative result as well as practical guidelines in the eMethods in Supplement 1.

### Statistical Analysis

All analyses were performed using R, version 4.3.1 statistical software (R Project for Statistical Computing) with the prodlim package for computing cumulative incidence with the presence of competing events. Data were analyzed from July 1, 2022, to January 31, 2023.

As sensitivity analyses, we conducted subgroup analyses by sex due to the demonstrated sex-based differential incidence of IPLC^29^ and smoking prevalence.^30,31^ Additional subgroup analyses were performed among patients with early-stage IPLC (ie, localized and regional stages based on the SEER summary stage) who may exhibit longer survival with longer time at risk of developing SPLC,^32^ as well as among patients with the most common IPLC histology (ie, adenocarcinoma) for those without a smoking history.^3^ In addition, we conducted Poisson regression analyses to evaluate the SIR differences by smoking status, adjusting for covariates (ie, IPLC stage and histology). We used the Bonferroni method to adjust for multiple testing for 6 comparisons across sex and IPLC stage and histology, with a 2-sided significance level of *P* = .008 (*P* = .05 / 6).

## Results

Among the 211 414 participants in MEC, the overall proportion of never-smokers was 44.7% compared with 55.3% of ever-smokers at enrollment (Table). Additional baseline characteristics of MEC have been previously reported.^20^ Of MEC participants, 7161 (3.96%) developed IPLC over 4 038 007 person-years. Of these participants, the mean (SD) age at cohort enrollment was 63.6 (7.7) years, 4031 (56.3%) were male, and 3131 (43.7%) were female. By race and ethnicity, the cohort diagnosed with IPLC consisted of 1852 participants (25.9%) who were African Americans, 1633 (22.8%) Japanese American, 1005 (14.0%) Latino, 578 (8.1%) Native Hawaiian, 1714 (23.9%) White, and 379 (5.3%) other race or ethnicity (Chinese, Filipino, Korean, or unspecified). In this IPLC population, 163 (2.28%) developed SPLC over 16 470 person-years, 6315 (88.1%) had a smoking history, 2864 (40.0%) had adenocarcinoma, and 2837 (39.6%) had early-stage lung cancer at diagnosis (Table).

**Table.** Characteristics of the Study Participants in the Multiethnic Cohort Study

Characteristic	No. (%)		
	Entire cohort (N = 211 414)	IPLC (n = 7161)	SPLC (n = 163)
Age at cohort enrollment, mean (SD), y	59.9 (8.8)	63.6 (7.7)	62.7 (7.1)
Sex			
Male	95 331 (45.1)	4031 (56.3)	84 (51.5)
Female	116 083 (54.9)	3131 (43.7)	79 (48.5)
Race and ethnicity			
African American	34 169 (16.2)	1852 (25.9)	35 (21.5)
Japanese American	56 272 (26.6)	1633 (22.8)	29 (23.9)
Latino	45 776 (21.7)	1005 (14.0)	20 (12.3)
Native Hawaiian	14 289 (6.8)	578 (8.1)	14 (8.6)
White	48 921 (23.1)	1714 (23.9)	47 (28.8)
Other^a^	11 987 (5.7)	379 (5.3)	8 (4.9)
Smoking history^b^			
Never	94 579 (44.7)	849 (11.9)	19 (11.7)
Former	82 856 (39.2)	3004 (41.9)	66 (40.5)
Current	33 979 (16.1)	3311 (46.2)	78 (47.9)
Prior history of cancer			
No	193 643 (91.6)	5308 (74.1)	112 (68.7)
Yes	17 769 (8.4)	1853 (25.9)	51 (31.3)
Family history of lung cancer			
No	198 643 (94.0)	6533 (91.2)	149 (91.4)
Yes	12 740 (6.0)	628 (8.8)	14 (8.6)
Smoking pack-years (excluding those who never smoked), mean (SD)	18.3 (15.9)	29.2 (17.4)	33.4 (17.7)
Missing	4765 (2.3)	158 (2.2)	2 (1.2)
Smoking pack-years (including those who never smoked [0 pack-years]), mean (SD)	9.9 (14.8)	25.6 (18.9)	29.4 (19.8)
Missing	4765 (2.3)	158 (2.2)	2 (1.2)
Age at IPLC diagnosis, mean (SD), y^c^	NA	74.3 (8.3)	72.1 (8.1)
IPLC stage at diagnosis^c^			
Early (localized and regional SEER summary stage)	NA	2837 (39.6)	145 (89.0)
Advanced (distant SEER summary stage)	NA	3872 (54.1)	16 (9.8)
Missing	NA	452 (6.3)	2 (1.2)
IPLC histology at diagnosis^c^			
Adenocarcinoma	NA	2864 (40.0)	97 (59.5)
Squamous cell	NA	1416 (19.8)	34 (20.9)
Large cell	NA	207 (2.9)	9 (5.5)
Non–small cell lung cancer or NOS	NA	571 (8.0)	4 (2.5)
Small cell	NA	737 (10.3)	3 (1.8)
Other	NA	1365 (19.1)	16 (9.8)

Abbreviations: IPLC, initial primary lung cancer; NA, not applicable; NOS, not otherwise specified; SPLC, second primary lung cancer.

^a^ Other racial and ethnic group includes Chinese, Filipino, Korean, or unspecified.

^b^ Smoking history data were collected at cohort enrollment.

^c^ Tumor characteristics data were derived via linkage to the Surveillance, Epidemiology, and End Results registry. We tabulated the characteristics of initial lung cancer for both IPLC and SPLC cases.

**Figure 1.**
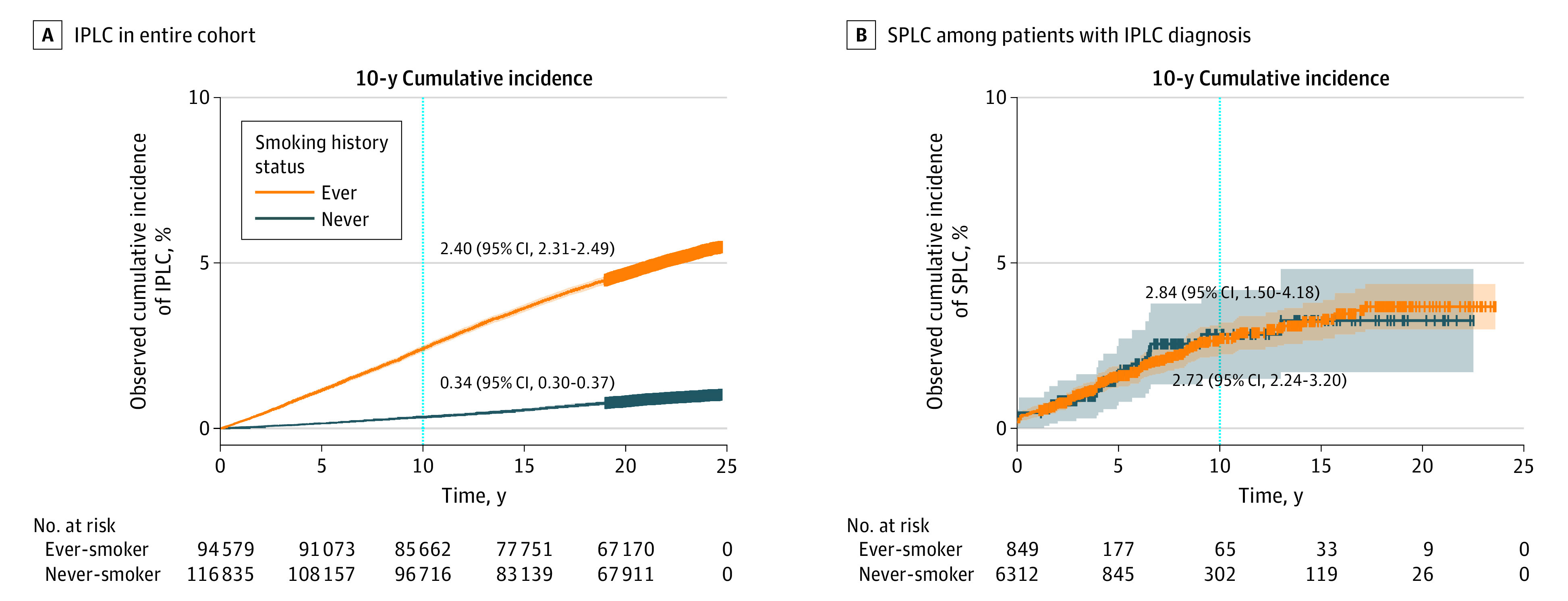
Cumulative Incidence of Initial Primary Lung Cancer (IPLC) in the Entire Multiethnic Cohort Study (N = 211 414) and Second Primary Lung Cancer (SPLC) Among the Patients With an IPLC Diagnosis (n = 7161) The dotted lines indicate 10 years from time of cohort enrollment (A) and 10 years from time of IPLC diagnosis (B). Gray and orange shading indicates the 95% CI of cumulative incidence of IPLC (A) and SPLC (B).

The overall 10-year cumulative incidence of IPLC in the entire cohort was 2.40% (95% CI, 2.31%-2.49%) among those who ever smoked, which was 7 times higher than the rate for those who never smoked (0.34%; 95% CI, 0.30%-0.37%) (Figure 1A). In contrast, the 10-year cumulative incidence of SPLC following IPLC diagnosis remained as high among patients with IPLC who never smoked (2.84%; 95% CI, 1.50%-4.18%) as those who ever smoked (2.72%; 95% CI, 2.24%-3.20%) (Figure 1B).

**Figure 2.**
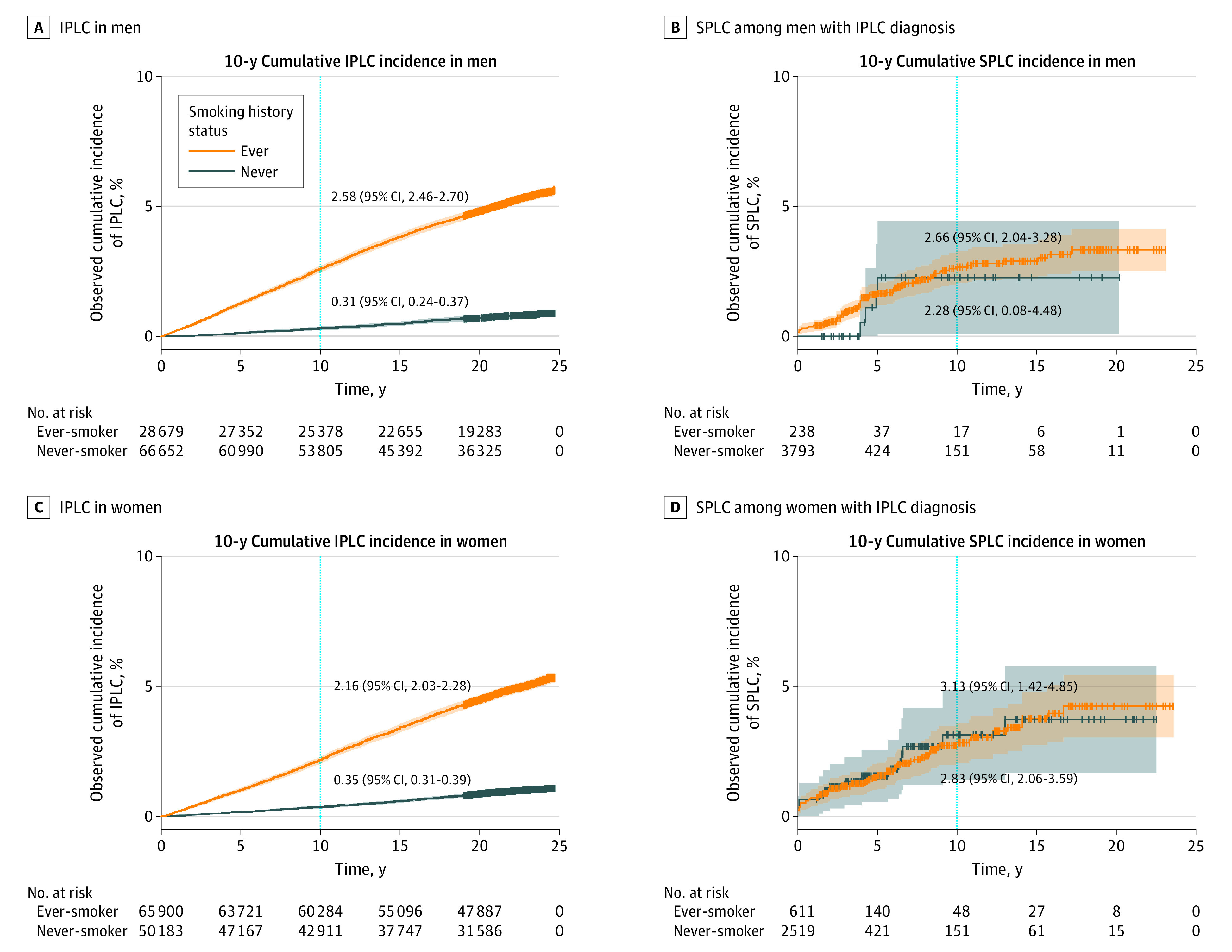
Cumulative Incidence of Initial Primary Lung Cancer (IPLC) and Second Primary Lung Cancer (SPLC) in Men and Women The dotted lines indicate 10 years from time of cohort enrollment (A and C) and 10 years from time of IPLC diagnosis (B and D). Gray and orange shading indicates the 95% CI of cumulative incidence of IPLC (A and C) and SPLC (B and D).

The overall SIR (ie, the ratio of SPLC to IPLC incidence) showed that the SPLC incidence (962.57 per 100 000 person-years) among patients with IPLC was 5.47 times higher than the IPLC incidence in the entire cohort (175.88 per 100 000 person-years) (SIR, 5.47; 95% CI, 4.67-6.38) (eTable 1 in Supplement 1). However, this SIR was substantially elevated (14.50; 95% CI, 8.73-22.65) among never-smokers (whose SPLC incidence was nearly as high as among ever-smokers despite their low IPLC incidence) compared with ever-smokers (3.50; 95% CI, 2.95-4.12) (eTable 1 in Supplement 1). We conducted a sensitivity analysis stratified by sex, given the differences in IPLC incidence^29^ and smoking prevalence^30,31^ by sex. The overall SIR among women (6.30; 95% CI, 4.99-7.85) was slightly higher than among men (4.69; 95% CI, 3.75-5.80) due to lower IPLC incidence among women, but the SIR difference by smoking history was similar across sexes (eTable 2 in Supplement 1; Figure 2). Given the high incidence of SPLC among patients with early-stage IPLC (ie, localized and regional),^14,22^ we conducted a subgroup analysis for those diagnosed with early-stage IPLC. The results were consistent with the primary analyses, with a broader gap in the SIR by smoking history due to a lower incidence of early-stage IPLC among never-smokers (eTable 3 and eFigure 1 in Supplement 1). Consistent results were also observed in the subgroup analysis of patients with IPLC of adenocarcinoma histology, which is the most common histologic subtype among never-smokers (eTable 4 and eFigure 2 in Supplement 1). Sensitivity analysis using Poisson regression after adjustment for covariates showed similar results, producing higher SIR estimates due to the adjusted survival based on IPLC stage and histology (eTable 5 in Supplement 1).

## Discussion

In this cohort study, we examined the patterns of SPLC incidence among lung cancer survivors who never vs ever smoked. The findings suggest that the incidence of SPLC remains as high after IPLC diagnosis among patients who never smoked as in those with a smoking history. We used data from MEC, a large, ethnically diverse cohort from California and Hawaii with a long follow-up that would allow the detection of IPLC through SPLC. When examining SPLC risk by smoking history in subgroups stratified by sex, early-stage IPLC, and adenocarcinoma histology, our results remained consistent.

Though still poorly understood, interest in risk factors for lung cancer among never-smokers has risen as smoking rates have decreased over the past decades. Radon,^5^ secondhand smoke,^6^ and occupational exposures (eg, asbestos)^33^ have each been associated with risk of IPLC among individuals without a smoking history, among other risk factors.^2,10,34,35^ Additionally, a particularly high incidence of IPLC has been shown in never-smoking Asian women,^36,37^ which may be relevant to this study given the racial diversity of the MEC. However, we were unable to obtain robust SIR estimates in our exploratory analysis stratified by sex and race combined due to a limited number of SPLC cases in the subgroup.

With therapeutic improvements and screening guidelines for IPLC, the number of SPLC cases among lung cancer survivors has gradually increased, but understanding of the risk factors for SPLC is still nascent. Although prior studies have identified tobacco smoking,^14^ IPLC stage and histology,^17,18^ or surgical resection for IPLC^15^ as potential risk factors for SPLC, little is known about risk factors specific to never-smokers. Casal-Mouriño et al^32^ suggested that median survival is longer among never-smokers than ever-smokers, which could increase the time at risk for SPLC for patients without a smoking history and may contribute to the high incidence of SPLC comparable to those with a smoking history. Other potential risk factors for SPLC may include underlying genetic susceptibility of the host,^2^ environmental factors,^2^ and long-term effects of cancer treatment.^38^ Of note, the recent treatment landscape of lung cancer evolved with the introduction of targeted therapies and immunotherapy, and the long-term effects of these novel therapies on SPLC have not been studied and warrant future investigation.

This study uncovers the elevated incidence ratio of SPLC vs IPLC among never-smokers and underscores the need for tailored surveillance strategies for individuals at high risk for SPLC, including never-smokers. While the risk of recurrence is highest in the first 2 to 4 years after curative treatment,^39,40^ SPLC exhibits a sustained risk for more than 10 years.^39,41^ The longer survival of never-smokers with IPLC may translate into an even longer time at risk for SPLC, thus requiring long-term follow-up. Current American Society of Clinical Oncology^42^ and National Comprehensive Cancer Network^43^ guidelines recommend low-dose computed tomography scans annually after 2 to 3 years following curative treatment for surveillance of secondary malignancies, but the optimal screening strategy is unclear beyond 5 years since IPLC diagnosis. As randomized clinical trials are unlikely in this setting where stopping screening or screening less than annually may result in potential harm, alternative novel methods such as trial emulation and simulation studies that leverage large health care utilization and outcomes data sets may be critical next steps to fill the evidence gap.

### Strengths and Limitations

To our knowledge, this study is among the first to evaluate the pattern of SPLC incidence by smoking history and to show a high incidence of SPLC among never-smokers using a population-based prospective cohort (MEC). Thus, this study directly addressed the lack of smoking status as a readily available variable in other large data sources, such as the Surveillance, Epidemiology, and End Results registry, which has prevented long-term IPLC and SPLC studies at the population level. Moreover, the racial and ethnic diversity of this data set increases the generalizability of the results from this study to a wider US demographic. Importantly, the use of large, prospective data with long-term follow-up from MEC, which followed through both IPLC and SPLC diagnoses, enabled an integrated analysis of SIR based on the use of a single large sample of the same individuals and allowed for subgroup analyses that showed consistent results regardless of sex, initial cancer stage of diagnosis, and IPLC histology.

This study also has several limitations. First, the SIR estimates were not age standardized as we followed the current practice of the MP-SIR approach.^27^ Therefore, there is a potential bias because the exposure time after IPLC may be at older ages and later in the calendar year than the exposure time before IPLC. Future methodological studies should consider age-standardized incidence. Second, we were unable to account for the long-term effects of novel therapies, such as targeted therapy and immunotherapy, as the time span of our data did not overlap with when these therapies were commonly used (after 2016). Thus, future studies should investigate the late effects of novel treatments using a more contemporary data set. Third, baseline smoking data were used in this study, which did not capture changes in smoking behavior after IPLC diagnosis. However, because the likelihood of starting to smoke following an IPLC diagnosis is minimal among never-smokers, smoking status (never vs ever smoking) is not expected to change after baseline. Nevertheless, smoking cessation following an IPLC diagnosis could reduce the cumulative incidence of SPLC in the long term, and SIR differences by smoking history may be larger than estimated if smoking cessation is considered.

## Conclusions

The findings of this cohort study show that the incidence of SPLC is as high among never-smokers as among those with a smoking history after an IPLC diagnosis, resulting in a substantially higher SIR among never-smokers than ever-smokers. While the reasons behind this observation are still poorly understood, the higher incidence of SPLC among never-smoking patients could be attributable to the longer survival reported in prior studies. Our findings highlight the critical need to identify risk factors for SPLC among never-smokers and to develop a targeted surveillance strategy for this patient population.

## Notes

Supplement 1. **eMethods.** Interpretation of Standardized Incidence Ratio (SIR) vs Cause-Specific Cox Regression
eReferences
**eTable 1. **The Standardized Incidence Ratio in the Overall Cohort and by Smoking History **eTable 2.** The Standardized Incidence Ratio in the Overall Cohort and by Smoking History, Stratified by Sex **eTable 3.** The Standardized Incidence Ratio in the Overall Cohort and by Smoking History Based on Early-Stage Cases of Initial Primary Lung Cancer **eTable 4.** The Standardized Incidence Ratio in the Overall Cohort and by Smoking History Based on Adenocarcinoma Initial Primary Lung Cancer **eTable 5.** Sensitivity Analysis of the Adjusted Standardized Incidence Ratio in the Overall Cohort and by Smoking History Using Poisson Regression **eFigure 1.** Cumulative Incidence of Early-Stage Initial Primary Lung Cancer in the General Population (n = 207 554), in A, and Cumulative Incidence of Second Primary Lung Cancer in Patients With Early-Stage Lung Cancer (n = 3289), in B **eFigure 2.** Cumulative Incidence of Adenocarcinoma Initial Primary Lung Cancer in the General Population (n = 207 179), in A, and Cumulative Incidence of Second Primary Lung Cancer in Patients With Early-Stage Lung Cancer (n = 2874), in B

Supplement 2.
Data Sharing Statement
