# Supplementary material for: Second Primary Lung Cancer Among Lung Cancer Survivors Who Never Smoked
Source: JAMA Netw Open. 2023 Nov 15;6(11):e2343278. doi: 10.1001/jamanetworkopen.2023.43278 (PMC10652150; doi:10.1001/jamanetworkopen.2023.43278)
Supplement: Supplement 1. — eMethods. Interpretation of Standardized Incidence Ratio (SIR) vs Cause-Specific Cox Regression eReferences eTable 1. The Standardized Incidence Ratio in the Overall Cohort and by Smoking History eTable 2. The Standardized Incidence Ratio in the Overall Cohort and by Smoking History, Stratified by Sex eTable 3. The Standardized Incidence Ratio in the Overall Cohort and by Smoking History Based on Early-Stage Cases of Initial Primary Lung Cancer eTable 4. The Standardized Incidence Ratio in the Overall Cohort and by Smoking History Based on Adenocarcinoma Initial Primary Lung Cancer eTable 5. Sensitivity Analysis of the Adjusted Standardized Incidence Ratio in the Overall Cohort and by Smoking History Using Poisson Regression eFigure 1. Cumulative Incidence of Early-Stage Initial Primary Lung Cancer in the General Population (n = 207 554), in A, and Cumulative Incidence of Second Primary Lung Cancer in Patients With Early-Stage Lung Cancer (n = 3289), in B eFigure 2. Cumulative Incidence of Adenocarcinoma Initial Primary Lung Cancer in the General Population (n = 207 179), in A, and Cumulative Incidence of Second Primary Lung Cancer in Patients With Early-Stage Lung Cancer (n = 2874), in B [file jamanetwopen-e2343278-s001.pdf]

Supplemental Online Content

Choi E, Su CC, Wu JT, et al. Second primary lung cancer among lung cancer survivors who never smoked. *JAMA Netw Open*. 2023;6(11):e2343278. doi:10.1001/jamanetworkopen.2023.43278

eMethods. Interpretation of Standardized Incidence Ratio (SIR) vs Cause-Specific Cox Regression

eReferences

eTable 1. The Standardized Incidence Ratio in the Overall Cohort and by Smoking History

eTable 2. The Standardized Incidence Ratio in the Overall Cohort and by Smoking History, Stratified by Sex

eTable 3. The Standardized Incidence Ratio in the Overall Cohort and by Smoking History Based on Early-Stage Cases of Initial Primary Lung Cancer

eTable 4. The Standardized Incidence Ratio in the Overall Cohort and by Smoking History Based on Adenocarcinoma Initial Primary Lung Cancer

eTable 5. Sensitivity Analysis of the Adjusted Standardized Incidence Ratio in the Overall Cohort and by Smoking History Using Poisson Regression

eFigure 1. Cumulative Incidence of Early-Stage Initial Primary Lung Cancer in the General Population (n = 207 554), in A, and Cumulative Incidence of Second Primary Lung Cancer in Patients With Early-Stage Lung Cancer (n = 3289), in B

eFigure 2. Cumulative Incidence of Adenocarcinoma Initial Primary Lung Cancer in the General Population (n = 207 179), in A, and Cumulative Incidence of Second Primary Lung Cancer in Patients With Early-Stage Lung Cancer (n = 2874), in B

This supplemental material has been provided by the authors to give readers additional information about their work.

© 2023 Choi E et al. *JAMA Network Open.*

Supplementary Method

eMethods. Interpretation of Standardized Incidence Ratio (SIR) vs Cause-specific Cox Regression

Standardized Incidence Ratio (SIR) refers to the high or low incidence of disease cases in a specific population of interest compared to the incidence of the disease cases in a referent population. In this present study, we use SIR to calculate the incidence of new primary lung cancer cases (i.e., second primary lung cancer [SPLC]) among patients who already have an initial primary lung cancer (IPLC) diagnosis compared to the incidence of IPLC cases in the general population without an existing lung cancer diagnosis.

$$SIR = \frac{SPLC\ incidence\ (ie, SPLC\ cases \div Person\ year\ of\ IPLC\ patients)}{IPLC\ incidence\ (ie, IPLC\ cases \div Person\ year\ of\ the\ entire\ cohort)}$$

Based on the formula, the overall SIR of 5.47 (95% CI: 4.67-6.38) in the entire cohort indicates that once individual is diagnosed with IPLC, the incidence of SPLC is 5.47 times higher. When stratified by smoking history, the SIR among never-smokers was 14.50 (8.73-22.65) and the SIR among ever-smokers was 3.50 (2.95-4.12). This substantially higher SIR in never-smokers results from the denominator of the SIR equation, with the incidence of IPLC in the never-smoking general population being substantially lower than the risk of IPLC in the ever-smoking general population (**Panel A** below). By contrast, in **Panel B** below, the cumulative incidence of SPLC after an initial IPLC diagnosis – the numerator of the SIR equation – is comparable among those who have ever or never smoked, albeit a slightly elevated risk at the 20-year mark since IPLC diagnosis among ever-smoking patients (as indicated by the red arrow). This reinforces our conclusion that patients without a smoking history have an elevated risk for SPLC as much as those with a smoking history once they have an IPLC diagnosis.

To further clarify the distinction between the SIR analysis and standard risk analysis (e.g., hazard ratio, relative risk, or odds ratio), we also conducted a comparative analysis using cause-specific Cox regression on the same study cohort (**Panel C**). As demonstrated in the table below, the number and proportion of those who have a competing event of death in IPLC patients before developing SPLC is notably high, implementing cause-specific Cox regression the recommended option over standard Cox regression.

Table. Proportion of competing-risk events by survival time since IPLC diagnosis in IPLC patients by a smoking history

	Censored (N, %)		SPLC (N, %)		Competing death (N, %)		Total
**Never-smoking IPLC patients**							
0-1 years	0	(0.0)	4	(21.1)	342	(56.4)	346
1-2 years	15	(6.7)	2	(10.5)	118	(19.5)	135
2-3 years	33	(14.7)	2	(10.5)	52	(8.6)	87
3-4 years	24	(10.7)	1	(5.3)	30	(5.0)	55
4-5 years	26	(11.6)	3	(15.8)	20	(3.3)	49
5+ years	126	(56.3)	7	(36.8)	44	(7.3)	177
Total	224	(100.0)	19	(100.0)	606	(100.0)	849
	Censored (N, %)		SPLC (N, %)		Competing death (N, %)		Total
**Ever-smoking IPLC patients**							
0-1 years	0	(0.0)	32	(22.2)	3405	(65.8)	3437
1-2 years	91	(9.1)	12	(8.3)	875	(16.9)	978
2-3 years	119	(11.9)	18	(12.5)	321	(6.2)	458
3-4 years	160	(16.1)	16	(11.1)	200	(3.9)	376
4-5 years	113	(11.3)	11	(7.6)	94	(1.8)	218
5+ years	513	(51.5)	55	(38.2)	277	(5.4)	845
Total	996	(100.0)	144	(100.0)	5172	(100.0)	6312

© 2023 Choi E et al. *JAMA Network Open.*

We observed 1.45 times increased risks of developing SPLC among ever-smoking IPLC patients compared to never-smoking patients, despite the lack of statistical significance due in part to the limited samples. When broken down by smoking status of former and current smokers versus never-smokers, the SPLC risk was 1.25 times higher among former smokers (aHR = 1.25 [0.74-2.09]) and 1.71 times higher current smokers (aHR = 1.71 [1.02-2.87]), which is in line with the cumulative incidence plot in **Panel B** showing a slightly elevated risk among ever-smokers indicated by red arrow. That is, tobacco smoking still plays a significant role in increasing the risk of SPLC among IPLC patients, similar with prior studies.[1-3]

Overall, the findings of this present study – by analysis using SIR or cause-specific Cox regression – demonstrate that smoking is a risk factor for SPLC in lung cancer patients. However, the elevated risk of SPLC following IPLC diagnosis persists in both those with and without a smoking history.

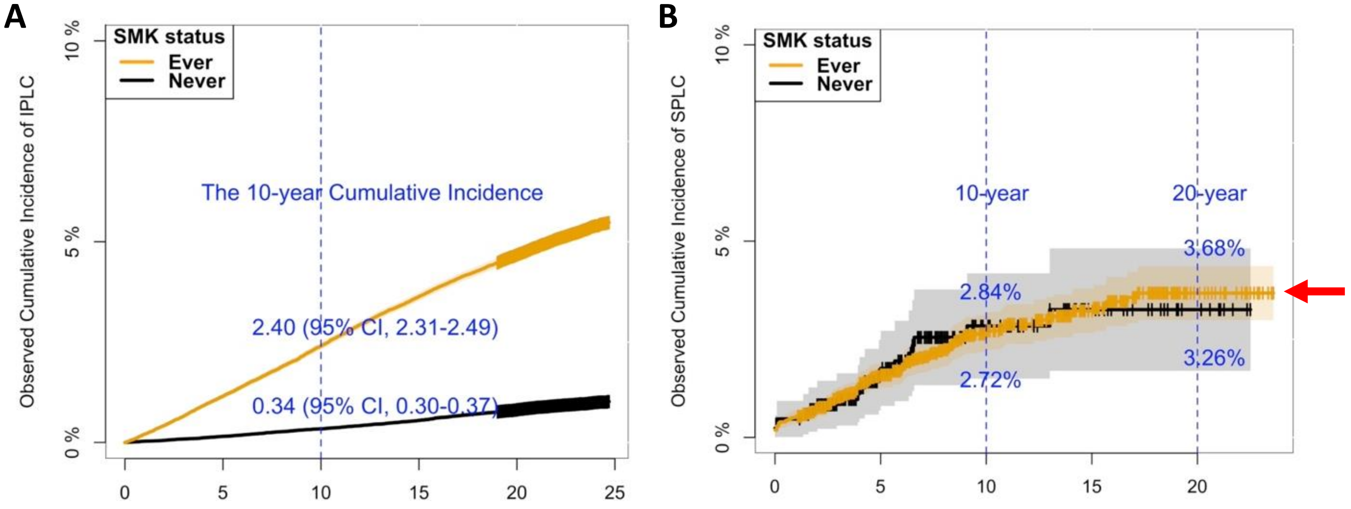

C

Cause-specific Cox regression to estimate the smoking effect on SPLC risk among IPLC patients

	Sample size	Adjusted hazard ratio	95% CI	*P*-Value
**Ever-smoking history at cohort enrollment**				
Never	19	1.00 (reference)		
Ever	144	1.45	(0.88 to 2.35)	0.13
**Smoking status at cohort enrollment**				
Never	19	1.00 (reference)		
Former	66	1.25	(0.74 to 2.09)	0.39
Current	78	1.71	(1.02 to 2.87)	0.04

Abbreviation: CI, confidence interval; IPLC, initial primary lung cancer, SPLC, second primary lung cancer
Note: Adjusted by age at IPLC diagnosis, IPLC histology, and IPLC stage

© 2023 Choi E et al. *JAMA Network Open.*

eReferences

1. Aredo JV, Luo SJ, Gardner RM, et al. Tobacco Smoking and Risk of Second Primary Lung Cancer. *J Thorac Oncol.* 2021;16(6):968-979.
2. Choi E, Luo SJ, Aredo JV, et al. The Survival Impact of Second Primary Lung Cancer in Patients With Lung Cancer. *J Natl Cancer Inst.* 2022;114(4):618-625.
3. Luo SJ, Choi E, Aredo JV, et al. Smoking Cessation After Lung Cancer Diagnosis and the Risk of Second Primary Lung Cancer: The Multiethnic Cohort Study. *JNCI Cancer Spectr.* 2021;5(5).

© 2023 Choi E et al. *JAMA Network Open.*

Supplementary Tables

eTable 1. The Standardized Incidence Ratio in the Overall Cohort and by Smoking History

Incidence (/100,000 person-years)	Overall cohort	Smoking History	
		Never	Ever
SPLC cases	163	19	144
SPLC incidence (95% CI)[1,3]	962.57 (826.58-1120.94)	701.72 (448.30-1098.39)	1021.08 (867.94-1201.25)
IPLC cases	7161	849	6312
IPLC incidence (95% CI)[1,4]	175.88 (171.88-179.98)	48.38 (45.36 -51.60)	291.88 (284.73-299.20)
SIR (95% CI)[2,5]	5.47 (4.67-6.38)	14.50 (8.73-22.65)	3.50 (2.95-4.12)

Abbreviations: IPLC, initial primary lung cancer; SIR, standardized incidence ratio, SPLC, second primary lung cancer.

Notes:

1) Confidence intervals for a Poisson rate using an exact method
2) Byar's approximation to the Poisson distribution
3) SPLC incidence was calculated as the incident SPLC cases over the person-year of the IPLC patients in the Multiethnic Cohort Study (MEC).
4) IPLC incidence was calculated as the incident IPLC cases over the person-year of the entire study population in the MEC.
5) An SIR is estimated as the SPLC incidence, divided by the IPLC incidence among the overall cohort and by smoking history.

© 2023 Choi E et al. *JAMA Network Open.*

eTable 2. The Standardized Incidence Ratio in the Overall Cohort and by Smoking History, Stratified by Sex

Incidence (/100,000 person-years)	Overall cohort	Smoking History	
		Never	Ever
Male			
SPLC cases	84	4	80
SPLC incidence (95% CI)[1,3]	1040.63 (842.27-1285.70)	640.95 (241.32-1702.41)	1074.71 (864.25-1336.43)
IPLC cases	4031	238	3793
IPLC incidence (95% CI)[1,4]	221.85 (215.02-228.90)	43.20 (38.20-49.09)	307.97 (298.15-318.13)
SIR (95% CI)[2,5]	4.69 (3.75-5.80)	14.80 (3.98-37.89)	3.49 (2.77-4.345)
Female			
SPLC cases	79	15	64
SPLC incidence (95% CI)[1,3]	890.69 (715.13-1109.35)	719.92 (434.81-1191.99)	961.14 (753.18-1226.51)
IPLC cases	3131	611	2519
IPLC incidence (95% CI)[1,4]	141.32 (136.60-146.21)	50.50 (46.85-54.44)	271.94 (261.69-282.59)
SIR (95% CI)[2,5]	6.30 (4.99-7.85)	14.26 (7.97-23.51)	3.53 (2.72-4.51)

Abbreviations: IPLC, initial primary lung cancer; SIR, standardized incidence ratio, SPLC, second primary lung cancer.

Notes:

1) Confidence intervals for a Poisson rate using an exact method
2) Byar's approximation to the Poisson distribution
3) SPLC incidence was calculated as the incident SPLC cases over the person-year of the IPLC patients in the Multiethnic Cohort Study (MEC).
4) IPLC incidence was calculated as the incident IPLC cases over the person-year of the entire study population in the MEC.
5) An SIR is estimated as the SPLC incidence, divided by the IPLC incidence among the overall cohort and by smoking history.

© 2023 Choi E et al. *JAMA Network Open.*

eTable 3. The Standardized Incidence Ratio in the Overall Cohort and by Smoking History Based on Early-Stage Cases of Initial Primary Lung Cancer

Incidence (/100,000 person-years)	Overall cohort	Smoking History	
		Never	Ever
SPLC cases	147	14	133
SPLC incidence (95% CI)[1,3]	1173.73 (1000.03-1377.61)	709.19 (420.80-1195.23)	1269.04 (1071.86-1502.50)
IPLC cases	2837	323	2514
IPLC incidence (95% CI)[1,4]	82.00 (79.27-84.83)	21.83 (19.83-24.04)	137.46 (132.54-142.56)
SIR (95% CI)[2,5]	14.31 (12.10-16.81)	32.48 (17.74-54.50)	9.23 (7.73-10.94)

Abbreviations: IPLC, initial primary lung cancer; SIR, standardized incidence ratio, SPLC, second primary lung cancer.

Notes:

1) Confidence intervals for a Poisson rate using an exact method
2) Byar's approximation to the Poisson distribution
3) SPLC incidence was calculated as the incident SPLC cases over the person-year of the IPLC patients in the Multiethnic Cohort Study (MEC).
4) IPLC incidence was calculated as the incident IPLC cases over the person-year of the entire study population in the MEC.
5) An SIR is estimated as the SPLC incidence, divided by the IPLC incidence among the overall cohort and by smoking history.

© 2023 Choi E et al. *JAMA Network Open.*

eTable 4. The Standardized Incidence Ratio in the Overall Cohort and by Smoking History Based on Adenocarcinoma Initial Primary Lung Cancer

Incidence (/100,000 person-years)	Overall cohort	Smoking History	
		Never	Ever
SPLC cases	97	15	82
SPLC incidence (95% CI)[1,3]	1143.71 (938.40-1393.95)	835.36 (504.67-1382.71)	1226.53 (989.22-1521.02)
IPLC cases	2864	522	2342
IPLC incidence (95% CI)[1,4]	73.13 (70.53-75.83)	30.77 (28.38-33.36)	111.73 (107.29-116.34)
SIR (95% CI)[2,5]	15.64 (12.68-19.08)	27.15 (15.18-44.78)	10.98 (8.73-13.63)

Abbreviations: IPLC, initial primary lung cancer; SIR, standardized incidence ratio, SPLC, second primary lung cancer.

Notes:

1) Confidence intervals for a Poisson rate using an exact method
2) Byar's approximation to the Poisson distribution
3) SPLC incidence was calculated as the incident SPLC cases over the person-year of the IPLC patients in the Multiethnic Cohort Study (MEC).
4) IPLC incidence was calculated as the incident IPLC cases over the person-year of the entire study population in the MEC.
5) An SIR is estimated as the SPLC incidence, divided by the IPLC incidence among the overall cohort and by smoking history.

© 2023 Choi E et al. *JAMA Network Open.*

eTable 5. Sensitivity Analysis of the Adjusted Standardized Incidence Ratio in the Overall Cohort and by Smoking History Using Poisson Regression[1]

Standardized Incidence Ratio SIR (95% Confidence Interval)	Smoking history	
	Never	Ever
Unadjusted estimate	14.51 (13.14-16.01)	3.49 (3.28-3.72)
Adjusted by IPLC stage	34.36 (31.95-36.95)	9.36 (8.89-9.86)
Adjusted by IPLC stage and IPLC histology[2]	69.40 (66.55-74.17)	13.32 (12.86-14.08)

Abbreviations: IPLC, initial primary lung cancer; SIR, standardized incidence ratio

Notes:

1) Poisson regression was implemented to fit the incidence of SPLC as of a function of offset of IPLC incidence with or without adjustment factors.

2) IPLC histology was classified as non-small cell lung cancer (NSCLC) vs. all others.

© 2023 Choi E et al. *JAMA Network Open.*

Supplementary Figures

eFigure 1. Cumulative Incidence of Early-Stage Initial Primary Lung Cancer in the General Population (n = 207 554), in A, and Cumulative Incidence of Second Primary Lung Cancer in Patients With Early-Stage Lung Cancer (n = 3289), in B

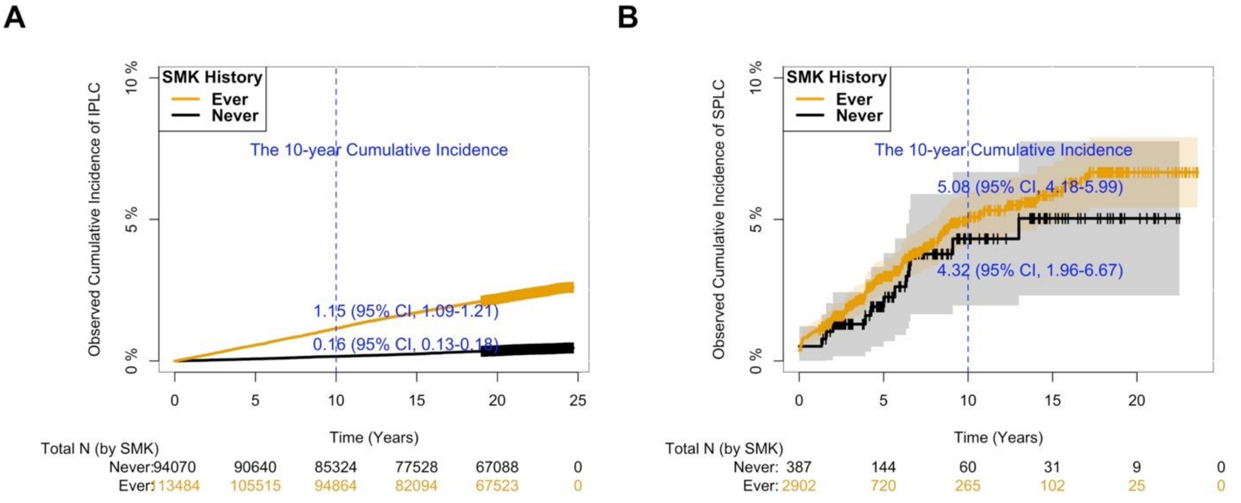

Abbreviations: IPLC, initial primary lung cancer; SMK, smoking history; SPLC, second primary lung cancer.
***Note*:**
a Dotted line in each panel indicates 10 years from time of cohort enrollment (A) and 10 years from time of IPLC diagnosis (B).
b Total N (by SMK) indicates number of individuals at risk by smoking history.

© 2023 Choi E et al. *JAMA Network Open.*

eFigure 2. Cumulative Incidence of Adenocarcinoma Initial Primary Lung Cancer in the General Population (n = 207 179), in A, and Cumulative Incidence of Second Primary Lung Cancer in Patients With Early-Stage Lung Cancer (n = 2874), in B

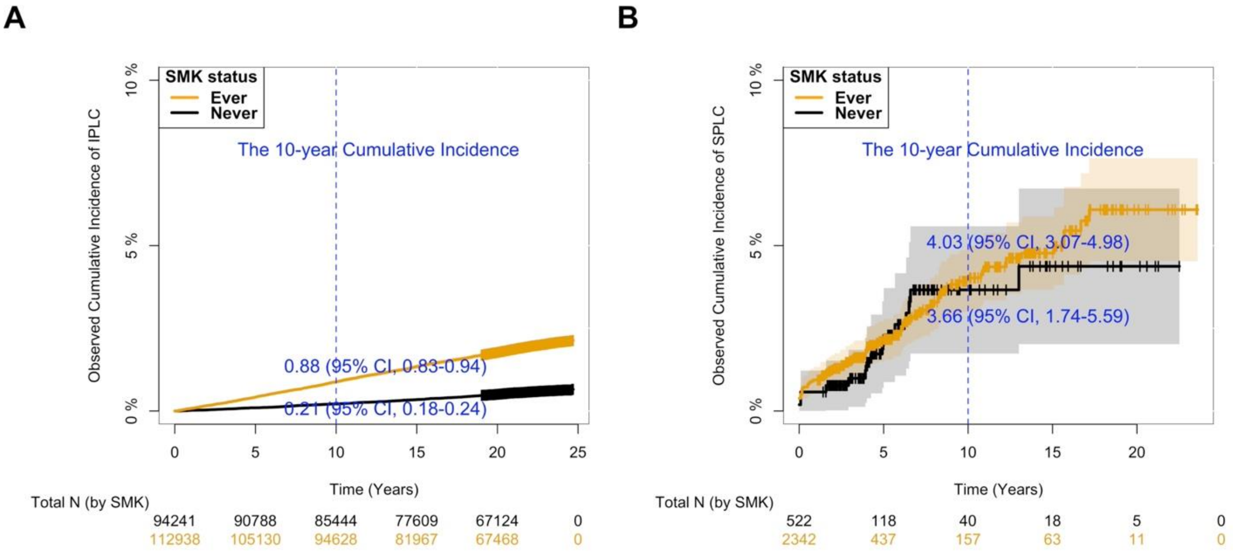

Abbreviations: IPLC, initial primary lung cancer; SMK, smoking history; SPLC, second primary lung cancer.
***Note*:**
a Dotted line in each panel indicates 10 years from time of cohort enrollment (A) and 10 years from time of IPLC diagnosis (B).
b Total N (by SMK) indicates number of individuals at risk by smoking history.

© 2023 Choi E et al. *JAMA Network Open.*
