# Supplementary material for: Second Primary Lung Cancer Among Lung Cancer Survivors Who Never Smoked
Source: JAMA Netw Open. 2023 Nov 15;6(11):e2343278. doi: 10.1001/jamanetworkopen.2023.43278 (PMC10652150; doi:10.1001/jamanetworkopen.2023.43278)
Supplement: Supplement 2. — Data Sharing Statement [file jamanetwopen-e2343278-s002.pdf]

Data Sharing Statement

Choi. Second Primary Lung Cancer Among Lung Cancer Survivors Who Never Smoked. *JAMA Netw Open*. Published November 15, 2023. doi:10.1001/jamanetworkopen.2023.43278

Data
Data available: No

Additional Information
Explanation for why data not available: The data underlying this analysis were provided by the Multiethnic Cohort Study (MEC) under data use agreement. Researchers interested in the MEC data may submit an inquiry online: https://www.uhcancercenter.org/for-researchers/mecdata-sharing.
